# Validation of administrative database codes for acute kidney injury in kidney transplant recipients

**DOI:** 10.1186/s40697-016-0108-7

**Published:** 2016-04-07

**Authors:** Amber O. Molnar, Carl van Walraven, Eric McArthur, Dean Fergusson, Amit X. Garg, Greg Knoll

**Affiliations:** Division of Nephrology, Department of Medicine, McMaster University, Hamilton, Ontario Canada; Clinical Epidemiology Program, Ottawa Hospital Research Institute, Ottawa, Ontario Canada; Institute for Clinical Evaluative Sciences, Toronto, Ontario Canada; Department of Medicine, University of Ottawa, Ottawa, Ontario Canada; Department of Epidemiology & Biostatistics, Western University, London, Ontario Canada; Division of Nephrology, Western University, London, Ontario Canada; Division of Nephrology, Department of Medicine, University of Ottawa, Ottawa, Ontario Canada; St. Joseph’s Healthcare, 50 Charlton Ave E, L8N 4A6 Hamilton, Ontario Canada

**Keywords:** Acute kidney injury, Transplant, Codes, Validity, Databases

## Abstract

**Background:**

Validation studies of acute kidney injury (AKI) diagnostic codes performed in the general population have shown poor sensitivity, but the accuracy of such codes in the kidney transplant population remains unknown.

**Objective:**

The objective of this study is to determine the accuracy of AKI diagnostic codes in kidney transplant recipients. We hypothesized that the sensitivity of diagnostic codes would be significantly greater in the kidney transplant population since these patients are closely followed by nephrologists and are more likely to have serum creatinine measured.

**Design:**

The design is a population-based retrospective cohort study using healthcare administrative and laboratory databases.

**Setting:**

The setting is in Southwestern Ontario and Ottawa, Ontario, Canada, from 2003 to 2012.

**Patients:**

We included first-time kidney transplant recipients admitted to hospital for whom serum creatinine was measured in hospital and within 6 months prior (*n* = 524).

**Methods:**

Patients meeting the Acute Kidney Injury Network (AKIN) classification serum creatinine change criteria were classified as having AKI. We determined the sensitivity, specificity, and negative and positive predictive values for the *ICD-10*-CA code for AKI when present as an admission diagnosis, most responsible diagnosis, or any diagnosis compared to a reference standard of AKI defined by the AKIN criteria (stage 1 or greater, stage 2 or greater, or stage 3).

**Results:**

Forty-five percent of included kidney transplant patients had a diagnosis of AKI. The most sensitive coding algorithm (reference standard AKIN stage 2 or greater, *ICD-*10 code present as any diagnosis) had a sensitivity of 42.1 % (95 % CI 31.7, 53.3), a specificity of 90.6 % (95 % CI 87.6, 93.0), and a positive likelihood ratio of 4.5. The median (IQR) rise in serum creatinine from baseline in patients with and without AKI codes was 104 (57 to 158) μmol/L and 16 (−3 to 41) μmol/L, respectively (Mann-Whitney test, *p* < 0.0001).

**Limitations:**

The low sensitivity of the AKI code may be due to an alternative diagnosis of acute rejection being assigned in certain cases. The cause of AKI could not be determined.

**Conclusions:**

Similar to the general population, the *ICD-10* N17x code misses many kidney transplant patients with AKI during their hospitalization. This makes the code unusable for studying the incidence and consequences of AKI in hospitalized kidney transplant patients.

**Electronic supplementary material:**

The online version of this article (doi:10.1186/s40697-016-0108-7) contains supplementary material, which is available to authorized users.

## Abrégé

**Mise en contexte:**

Les études de validation portant sur les codes diagnostiques pour l’identification de l’insuffisance rénale aiguë (IRA) menées dans la population générale ont révélé une piètre sensibilité de détection de ceux-ci. Toutefois, leur fiabilité chez les patients ayant subi une greffe du rein reste à démontrer.

**Objectif de l’étude:**

Cette étude visait à évaluer la précision des codes de diagnostic de l’IRA chez les patients ayant reçu une greffe rénale. Le postulat de départ était que la sensibilité de ces codes serait nettement supérieure au sein de cette population puisque ces patients bénéficient d’un suivi par un néphrologue et sont plus susceptibles de voir leur taux de créatinine mesuré régulièrement.

**Cadre et type d’étude:**

Il s’agit d’une étude de cohorte rétrospective, représentative de la population. Elle a été réalisée entre 2003 et 2012, à Ottawa et dans le sud-ouest de l’Ontario, à partir de bases de données de laboratoire.

**Participants:**

Un total de 524 receveurs d’une première greffe rénale ayant été hospitalisés et dont la créatinine a été mesurée au moment de l’hospitalisation et dans les 6 mois précédant celle-ci.

**Méthodologie:**

Les patients dont le taux de créatinine correspondait aux stades de classification de l’IRA du *Acute Kidney Injury Network* (AKIN) ont été considérés en insuffisance rénale aiguë. Des valeurs de spécificité et de sensibilité ainsi que des valeurs de prédiction positives et négatives pour le code *ICD-10*-CA en situation d’IRA ont été déterminées lorsque l’IRA constituait le diagnostic d’admission, le diagnostic le plus probable mis en cause, ou n’importe lequel des diagnostics évoqués, en comparaison avec les standards de référence définis par les critères de l’AKIN (stade 1 ou supérieur, stade 2 ou supérieur, ou stade 3).

**Résultats:**

Au total, 45 % des patients admis dans cette étude ont reçu un diagnostic d’IRA. L’algorithme de codage le plus sensible [stade 2 en référence aux standards AKIN avec pour tout diagnostic la présence du code *ICD-10*-CA] présentait une sensibilité de 42,1 % [95 % IC 31,7 à 53,3], une spécificité s’élevant à 90,6 % [95 % IC 87,6 à 93,0] et un rapport de vraisemblance positif de 4,5. La hausse médiane [étendue interquartile] de créatinine sérique par rapport aux valeurs de base était de 16 [-3 à 41] μmol/L pour les patients non codés et de 104 [57 à 158] μmol/L pour les patients chez qui on décelait le code *ICD-10*-CA [test de Mann-Whitney, *p* < 0,000 1].

**Limite de l’étude:**

Dans certains cas, la faible sensibilité du code *ICD-10*-CA pourrait s’expliquer par un code de rejet aigu. Les causes d’épisodes d’IRA n’ont pas pu être établies dans cette étude.

**Conclusions:**

À l’instar de l’ensemble de la population, le code *ICD-10* N17x sous-estime l’incidence de l’IRA chez les greffés rénaux lors d’une hospitalisation. Ceci rend l’utilisation de ce code peu propice à des fins d’études sur l’incidence et les conséquences de l’IRA chez les patients hospitalisés qui ont subi une greffe du rein.

## What was known before

Validation studies performed in the general population demonstrate that acute kidney injury (AKI) diagnostic codes have low sensitivity but high specificity. The accuracy of AKI diagnostic codes in the kidney transplant population has not been studied.

## What this adds

The *ICD-10* N17x diagnostic code for AKI has a low sensitivity in the kidney transplant population, making the code unusable for studying the incidence and consequences of AKI in hospitalized kidney transplant patients. These results help inform the conduct of future studies in the kidney transplant population utilizing administrative data.

## Background

Health administrative databases house an enormous amount of data that might permit the conduct of large observational studies in an efficient, relatively low-cost manner [1, 2]. However, researchers utilizing such databases must be aware of the limitations of the data and the potential for biased results [3–5]. In particular, the validity of studies for which key exposures or outcomes are identified with diagnostic or procedural codes depends upon the accuracy of such codes [5, 6]. When used for clinical research, the accuracy of diagnostic and procedural codes for the entities they are supposed to represent should be determined.

The accuracy of diagnostic codes for acute kidney injury (AKI) has been measured in the general population, showing a very low sensitivity (approximately 30 %) but a notably high specificity (generally >95 %) [7]. A recent study examined the incidence and outcomes associated with AKI in the kidney transplant population, with AKI being defined using diagnostic codes [8]. However, the accuracy of diagnostic codes for AKI has never been determined in the kidney transplant population.

This study measured the accuracy of the *International Classification of Diseases, Tenth Revision* (*ICD-10*) code N17x for AKI in kidney transplant recipients admitted to hospital. We hypothesized that the *ICD-10* code would more accurately identify AKI in the kidney transplant population compared to the general population because kidney transplant patients have a higher prevalence of AKI, are more likely to have their kidney function followed closely, and are more likely to have a nephrologist involved in their care during a hospital admission [8–10].

## Methods

### Study design and setting

We conducted a population-based retrospective validation study in the province of Ontario, Canada, using Ontario’s population-based health administrative databases at the Institute for Clinical Evaluative Sciences (ICES) and laboratory data from Southwestern Ontario and Ottawa, Ontario. Residents of Ontario have universal access to hospital care and physician services under a single provincial payer system, resulting in a comprehensive repository of health administrative data. The availability of laboratory data was limited to Southwestern Ontario and Ottawa, Ontario. The study was conducted according to a pre-specified protocol that was approved by the Ottawa Hospital Research Ethics Board. The reporting of this study follows guidelines set out for studies assessing diagnostic accuracy (Appendix 1) [11].

### Data sources

We created our study’s analytical dataset using seven databases that were linked using encrypted unique identifiers. We identified kidney transplant recipients using the Canadian Organ Replacement Register (CORR), which captures data on every kidney transplant in the province of Ontario [12]. Laboratory data were obtained from the Ottawa Hospital Data Warehouse (OHDW) for Ottawa patients and from Cerner and Gamma-Dynacare for Southwestern Ontario patients. OHDW houses inpatient and outpatient lab information for individuals who had bloodwork drawn at one of three hospitals in Ottawa, Ontario. Cerner is a hospital network in Southwestern Ontario, housing inpatient and outpatient laboratory data from 12 hospitals. Gamma-Dynacare is a laboratory service provider that contains outpatient lab information for individuals who had bloodwork drawn at any of their 148 collection sites in Ontario. Demographics and vital status information were obtained from the Ontario Registered Persons Database (which records the sex, birthdate, and death date of all Ontarians) and CORR. Diagnostic and procedural information from all hospitalizations were determined using the Canadian Institute for Health Information Discharge Abstract Database (CIHI-DAD), which captures data on every hospitalization in the province of Ontario. Information was also obtained from the Ontario Health Insurance Plan database, which contains all health claims for inpatient and outpatient physician services. We have previously used these databases to research kidney health outcomes and health services [13–15].

### Study cohort

We included patients with the following characteristics: (a) first kidney-only transplant recipients; (b) hospitalized 6 months or later following kidney transplantation; (c) having at least one serum creatinine value measured during the hospital admission; (d) discharged from hospital prior to the end date of laboratory data availability; and (e) serum creatinine data available anytime between 2 weeks to 6 months prior to the admission date to determine baseline creatinine. Hospital admissions less than 6 months post transplant were excluded to eliminate as much as possible AKI secondary to post-operative complications, delayed graft function, and early acute rejection. Hospital admissions occurring between April 1, 2003 and December 31, 2012 (Ottawa) and March 31, 2012 (Southwestern Ontario) were eligible for inclusion. Hospital admissions with an admission date prior to April 1st, 2003 were excluded due to the use of *ICD-9* diagnostic codes prior to this date. Originally, *ICD-9* codes were included as a separate analysis, but this analysis had to be suppressed (as per ICES privacy regulations), due to the presence of too many small cells (total *n* = 118 patients). A look-back period of 3 years from the date of hospital admission was used to determine comorbidities. Codes used to define comorbidities of interest are outlined in Appendix 2. When multiple eligible hospital admissions were available for a patient, one was selected at random in order to avoid clustering in the analysis.

### Criteria for AKI

We used the Acute Kidney Injury Network (AKIN) staging system to define AKI [16]. AKIN stage 1 is defined by an increase in serum creatinine ≥26.4 μmol/L (0.3 mg/dL) or a 1.5- to 2-fold increase from baseline. AKIN stage 2 is defined by a >2- to 3-fold increase in serum creatinine from baseline. AKIN stage 3 is defined by an increase in serum creatinine >3-fold from baseline, or a serum creainine >354 μmol/L, with an acute increase of at least 44 μmol/L (0.5 mg/dL) [16]. The urine output criterion for the AKIN staging system was not used as these data were not available from our databases. The peak creatinine during a hospital admission was used to define the presence or absence of AKI and the AKIN stage. If multiple baseline creatinine values were available, the most recent value was used, except if drawn less than 2 weeks prior to admission. Creatinine values drawn very close to admission were excluded due to a heightened chance of the patient being unwell at the time of the bloodwork; the result may therefore not reflect a true baseline value but possibly the beginning of the AKI episode.

### ICD-10 code for AKI

Trained coders review all charts to record appropriate diagnosis codes and their associated attributes following a discharge from hospital. Coders follow the Canadian Coding Standards developed by CIHI [17]. According to CIHI’s guidelines, the coders are not permitted to interpret laboratory tests; however, they can record a condition based on laboratory measurements if a physician documents the condition in the patient’s chart. For hospitalization records (included in the CIHI-DAD), coders may record up to 25 conditions using *ICD-10* diagnostic codes. They must also indicate the diagnosis type. A diagnosis type “M” is the main or most responsible diagnosis, which is the condition that contributed most to the hospital length of stay or used the greatest amount of resources. An admission diagnosis is any condition that existed prior to the admission and was treated during the hospital stay [17].

In our study, we tested the accuracy of the *ICD-10* code N17x, which defines “acute renal failure,” when present as diagnosis type “M” or most responsible/main diagnosis, admission type diagnosis, or any diagnosis type (present in any one of the 25 potential diagnosis fields).

### Statistical analysis

We calculated the sensitivity, specificity, positive predictive value (PPV), and negative predictive value of the *ICD-10* N17x code compared to a reference standard of changes in serum creatinine using the AKIN staging system for AKI (formulas and a sample 2 × 2 table are presented in Appendix 3). We calculated 95 % confidence intervals for single proportions using the Wilson Score method [18]. We calculated the positive likelihood ratio using the sensitivity and specificity (Appendix 3). We also compared the change in serum creatinine between patients who were coded positive or negative for N17x. The difference in the distribution of change in creatinine between code negative and code positive patients was formally tested using the Mann-Whitney test. We conducted all analyses using the SAS software, version 9.4 (SAS institute Inc., Cary, NC, USA).

## Results

We identified a total of 524 kidney transplant patients with eligible hospital admissions from 2003 to 2012 that met our inclusion criteria. Patient selection is outlined in Additional file 1: Figure S1. Baseline characteristics are outlined in Table 1. The mean (standard deviation, SD) age was 57.7 (12.1) years. The median (interquartile range, IQR) time from kidney transplant to the index hospital admission was 3.5 (1.5, 7.1) years. The baseline serum creatinine was measured a median (IQR) of 34 (22, 68) days prior to hospital admission. The peak creatinine was measured a median (IQR) of 1 (0, 2) day post admission. AKI (based on AKIN criteria) occurred in 45.0 % of the cohort, and 14.1 % of the cohort was coded with *ICD-10* N17x. Of the patients with AKI, most (67.8 %) had mild disease (AKIN stage 1).

**Table 1:** Baseline characteristics

Total *n* = 524	
Demographics	
Mean age (SD), years	57.7 (12.1)
Age (*n* (%))	
18 to <35	23 (4.4)
35 to <60	262 (50)
60 to <70	151 (28.8)
≥70	88 (16.8)
Women (*n* (%))	185 (35.3)
Race (*n* (%))	
Caucasian	374 (71.4)
Asian	15 (2.9)
Black	19 (3.6)
Indian subcontinent	12 (2.3)
Aboriginal	9 (1.7)
Other	16 (3.1)
Unknown	34 (6.5)
Missing	45 (8.6)
Income quintile (*n* (%))	
One (lowest)	114 (21.8)
Two	106 (20.2)
Three	102 (19.5)
Four	83 (15.8)
Five (highest)	119 (22.7)
Rural location (*n* (%))	102 (19.5)
Year of cohort entry (*n* (%))	
2003–2006	149 (28.4)
2007–2009	159 (30.4)
2010–2012	216 (41.2)
Time since transplant, median (IQR), years	3.5 (1.5, 7.1)
Year of kidney transplant (n (%))	
1988–1992	18 (3.4)
1993–1997	39 (7.45)
1998–2002	147 (28.1)
2003–2007	205 (39.1)
2008–2012	115 (21.9)
Time on dialysis prior to transplant, median (IQR), years	2.0 (0.9, 3.5)
Time on dialysis prior to transplant (*n* (%)), years	
0 to <1	121 (23.1)
1 to <2	118 (22.5)
2 to <3	82 (15.7)
3 to <4	57 (10.9)
≥4	98 (18.7)
Cause of end stage renal disease (*n* (%))	
Glomerulonephritis	148 (28.2)
Hypertension	49 (9.4)
Pyelonephritis/interstitial	35 (6.7)
Cystic kidney disease	61 (11.6)
Diabetes	108 (20.6)
Other	29 (5.5)
Unknown	94 (17.9)
Type of donor (*n* (%))	
Deceased	347 (66.2)
Living	165 (31.5)
Missing	12 (2.3)
Comorbidities (*n* (%))	
Coronary artery disease	156 (29.8)
Diabetes	243 (46.4)
Hypertension	489 (93.3)
Congestive heart failure	88 (16.8)
Chronic liver disease	36 (6.9)
Chronic obstructive pulmonary disease	20 (3.8)
Peripheral vascular disease	28 (5.3)
Stroke/transient ischemic attack	12 (2.3)
Baseline laboratory measurements	
Serum creatinine, median (IQR), umol/L	133.0 (103.0, 173.9)
eGFR, median (IQR), ml/min/1.73 m^2a^	46.1 (33.4, 62.8)
eGFR category (*n* (%)), ml/min/1.73 m^2^	
<15	21 (4.0)
15 to <30	84 (16.0)
30 to <45	146 (27.9)
45 to <60	116 (22.1)
≥60	157 (30.0)
AKI definitions (*n* (%))^b^	
Any AKI	236 (45.0)
AKIN stage 1	160 (30.5)
AKIN stage 2	21 (4.0)
AKIN stage 3	55 (10.5)

^a^Estimated glomerular filtration rate (eGFR) was calculated using the CKD-EPI equation [32]

^b^Defined by serum creatinine

The diagnostic performance of the various coding algorithms is presented in Table 2. The diagnostic code type of “all diagnoses” performed the best. Compared to a reference standard of AKIN stage 1 or greater, the *ICD-10* N17x code for AKI showed a sensitivity of 28.0 % (95 % CI 22.6, 34.0) and specificity of 97.2 % (95 % CI 94.6, 98.6). Compared to a reference standard of AKIN stage 2 or greater, the *ICD-10* code showed a sensitivity of 42.1 % (95 % CI 31.7, 53.3) and a specificity of 90.6 % (95 % CI 87.6, 93.0). Overall, specificity was high, >90 % for most code types and definitions of AKI. The positive predictive value decreased significantly with increasing severity of AKI: AKIN stage 1 or greater 89.2 % (95 % CI 80.1, 94.4); AKIN stage 2 or greater 43.2 % (95 % CI 32.6, 54.6); and AKIN stage 3 29.7 % (95 % CI 20.5, 40.9) (Table 2).

**Table 2:** Diagnostic performance characteristics of three different algorithms for *ICD-10* code N17x using the AKIN staging system for AKI as the reference standard

Diagnostic coding algorithm	AKIN stage	Diagnostic performance characteristics (95 % CI)
All diagnoses	AKIN stage 1 or greater	Sn = 28.0 (22.6, 34.0)^a^
		Sp = 97.2 (94.6, 98.6)
		PPV = 89.2 (80.1, 94.4)
		NPV = 62.2 (57.7, 66.6)
		LR+ = 10.0
	AKIN stage 2 or greater	Sn = 42.1 (31.7, 53.3)
		Sp = 90.6 (87.6, 93.0)
		PPV = 43.2 (32.6, 54.6)
		NPV = 90.2 (87.1, 92.6)
		LR+ = 4.5
	AKIN stage 3	Sn = 40.0 (28.1, 53.2)
		Sp = 88.9 (85.8, 91.4)
		PPV = 29.7 (20.5, 40.9)
		NPV = 92.7 (89.9, 94.7)
		LR+ = 3.6
Admission diagnosis	AKIN stage 1 or greater	Sn = 20.3 (15.7, 25.9)
		Sp = 97.9 (95.5, 99.0)
		PPV = 88.9 (77.8, 94.8)
		NPV = 60.0 (55.5, 64.3)
		LR+ = 9.7
	AKIN stage 2 or greater	Sn = 34.2 (24.5, 45.4)
		Sp = 93.8 (91.1, 95.6)
		PPV = 48.2 (35.4, 61.2)
		NPV = 89.4 (86.3, 91.8)
		LR+ = 5.5
	AKIN stage 3	Sn = 32.7 (21.8, 45.9)
		Sp = 92.3 (89.6, 94.4)
		PPV = 33.3 (22.2, 46.6)
		NPV = 92.1 (89.3, 94.2)
		LR+ = 4.2
Main diagnosis/most responsible diagnosis	AKIN stage 1 or greater^b^	
	AKIN stage 2 or greater	Sn = 17.1 (10.3, 27.1)
		Sp = 97.3 (95.4, 98.5)
		PPV = 52.0 (33.5, 70.0)
		NPV = 87.4 (84.2, 90.0)
		LR+ = 6.3
	AKIN stage 3	Sn = 18.2 (10.2, 30.3)
		Sp = 96.8 (94.8, 98.1)
		PPV = 40.0 (23.4, 59.3)
		NPV = 91.0 (88.2, 93.2)
		LR+ = 5.7

^a^Used the Wilson 95 % confidence interval

^b^Results suppressed as per ICES privacy regulations due to the presence of small cells

*Sn* sensitivity, *Sp* specificity, *PPV* positive predictive value, *NPV* negative predictive value, *LR+* positive likelihood ratio

The absolute and relative changes in serum creatinine for patients that were coded positive and negative for AKI are presented in Table 3 and Additional file 1: Figures S2 and 3. In patients who were code positive and code negative for AKI, the median (IQR) absolute rise in serum creatinine from baseline was 104 (57 to 158) μmol/L and 16 (−3 to 41) μmol/L, respectively. The median (IQR) percent relative change was 56.9 (35 to 111) and 12.9 (−2.2 to 31), for code positive and code negative patients, respectively. The difference in the distribution of the absolute and relative changes in serum creatinine between code negative and code positive patients was statistically significant when the distributions were compared using the Mann-Whitney test (*p* < 0.0001).

**Table 3:** Change in serum creatinine from baseline in all patients with and without an ICD-10 N17x code for AKI (referred to as code positive or code negative)

Diagnostic coding algorithm	Code	Number	Absolute change (μmol/L) Median (IQR)	Relative change (%)^a^
All diagnoses	+	74	104.2 (57.0 to 158.0)	56.9 (35.0 to 111.4)
	−	450	16.0 (−3.0 to 41.0)	12.9 (−2.2 to 30.5)
Admission diagnosis	+	54	109.0 (62.0 to 161.0)	64.0 (35.0 to 122.1)
	−	470	18.0 (−2.0 to 45.0)	13.8 (−1.5 to 32.5)
Main diagnosis/most responsible diagnosis	+	25	128.0 (57.0 to 160.0)	71.3 (27.4 to 122.1)
	−	499	19.0 (−1.0 to 51.0)	15.3 (−0.7 to 37.4)

^a^Relative change = (peak serum creatinine − baseline serum creatinine)/baseline serum creatinine × 100

## Discussion

This study measured the accuracy of the *ICD-10* N17x code for the diagnosis of AKI in the kidney transplant population. The best performing code type was “all diagnoses” (i.e., present in any diagnostic field during a hospital admission). All code types, when compared to all definitions of AKI, showed a low to moderate sensitivity but high specificity. For AKI AKIN stage 1 or greater (any type of AKI), the positive predictive value of the code was high at almost 90 %. This suggests that the code would be reasonable for cohort selection if only kidney transplant patients with AKI were of interest. However, one would have to be satisfied that there was no differential misclassification, given the low sensitivity of the code, resulting in the exclusion of many patients who truly have AKI. Also, if only patients with more severe forms of AKI (AKIN stage 2 or 3) were of interest, the code would not be appropriate for cohort creation given the low prevalence of this disease stage with a resultant low positive predictive value.

The code performed poorly when a code type of “main or most responsible diagnosis” was used. This could be due to the fact that AKI often occurs in the setting of another illness, such as an infection [9, 19], which may be coded as the main diagnosis as opposed to AKI. The positive predictive value of the code was quite variable depending on the reference standard used. A low positive predictive value for severe AKI (AKIN stage 3) was found with all code types. This is likely due to the very low prevalence of AKIN stage 3 (10.5 %) in our cohort. The positive predictive value of a test (in this case the code) is known to vary significantly depending on the prevalence of the disease [20]. Specificity was quite high for all code types; however, it was slightly lower for code types with a higher sensitivity. Most code types were more sensitive (less false negatives) when a higher stage of AKI was used as the reference standard. This is to be expected because severe AKI is more clinically apparent and therefore more likely to be recorded in the chart.

The sensitivity of the code was lower than expected. When examined in healthy elderly and elderly patients with chronic kidney disease (CKD) (both at higher risk for AKI [21–23], similar to kidney transplant patients), the *ICD-10* code for AKI had a sensitivity of 62 and 76 % respectively, compared to a reference of at least a doubling in serum creatinine [24]. One possible reason for the lower than expected sensitivity of the code is that acute rejection (a cause of AKI), has its own diagnostic code. Although we could not verify the true incidence of acute rejection in our study, it should be very low given that the median time from transplant to index admission was 3.5 years, and acute rejection is very infrequent beyond the first year [25]. Nonetheless, a small proportion of AKI episodes (determined based on the reference standard of a rise in creatinine) were likely assigned a code for acute rejection as opposed to AKI. The specificity of the code in the kidney transplant population was slightly lower than in the general or elderly population [7], suggesting that kidney transplant patients are more likely to have a code assigned for AKI when their kidney function is actually stable. Overall, the code demonstrated limited sensitivity; however, a high positive predictive value for any AKI was found. The code was also able to distinguish two populations with significantly different rises in serum creatinine.

To our knowledge, this is the first study to measure the accuracy of the *ICD-10* diagnostic code for AKI in the kidney transplant population. Prior studies have examined the accuracy of AKI codes, mostly *ICD-9*, in the general, elderly, and elderly CKD populations [7, 24, 26]. We studied transplant patients from two healthcare regions in the province of Ontario, making the sample more representative and thus generalizable. We had serum creatinine values available, making it possible to compare the administrative diagnostic code to the gold standard for diagnosing AKI, as opposed to relying on chart re-abstraction.

Limitations to our study should be noted. First, for the reference standard used to define AKI, we adapted the creatinine-based component of the AKIN classification system, which defines AKI using both serum creatinine and urine output measurements [16]. It is also recommended that the AKIN classification system be applied only after a patient has achieved an optimal state of hydration. Unfortunately, urine output measurements and clinical data, such as hydration status and the administration of intravenous fluids, were not available in the administrative datasets that we used for this study. However, even if urine output data were available, urine output measurements are difficult to obtain and are poorly documented outside the intensive care setting. In addition, the sole use of serum creatinine is a commonly accepted method of defining AKI, both clinically and for research purposes [9, 27, 28]. Second, there is no consensus definition for AKI that has been validated in the kidney transplant population [29, 30]. However, all established classification systems apply similar serum creatinine and urine output criteria [30, 31]; AKI is defined similarly for transplant and non-transplant patients in the clinical setting; and the AKIN staging system was used to define AKI and correlated with poor outcomes in a prior study of kidney transplant patients [9]. Third, we did not specify a timeline of <48 h within which AKI had to occur (as specified in the AKIN criteria). The use of stringent timelines would likely diminish the accuracy of the AKI code since these timelines are not applied in the clinical setting. Finally, data on the cause of AKI was not available. The accuracy of administrative coding for AKI may differ depending on the cause, especially in transplantation, where a diagnosis of acute rejection may be coded preferentially over a diagnosis of AKI.

## Conclusions

In summary, our study demonstrates that identifying AKI in kidney transplant patients using administrative diagnostic codes will result in an underestimation of the true incidence and misclassification of patients with AKI. This limitation makes the code ineffectual for determining the incidence and consequences of AKI in hospitalized kidney transplant patients.

## Appendix 1

**Table 4:** STARD (STAndards for the Reporting of Diagnostic accuracy studies) checklist

Section and topic	Item #		Page
Title/Abstract/Keywords	1	Identify the article as a study of diagnostic accuracy (recommend MeSH heading “sensitivity and specificity”).	1–3
Introduction	2	State the research questions or study aims, such as estimating diagnostic accuracy or comparing accuracy between tests or across participant groups.	4–5
Methods			
Participants	3	The study population: The inclusion and exclusion criteria, setting and locations where data were collected.	5–7
	4	Participant recruitment: Was recruitment based on presenting symptoms, results from previous tests, or the fact that the participants had received the index tests or the reference standard?	5–7
	5	Participant sampling: Was the study population a consecutive series of participants defined by the selection criteria in item 3 and 4? If not, specify how participants were further selected.	7
	6	Data collection: Was data collection planned before the index test and reference standard were performed (prospective study) or after (retrospective study)?	7
Test methods	7	The reference standard and its rationale.	7–8
	8	Technical specifications of material and methods involved including how and when measurements were taken, and/or cite references for index tests and reference standard.	8–9
	9	Definition of and rationale for the units, cutoffs and/or categories of the results of the index tests and the reference standard.	7–9
	10	The number, training, and expertise of the persons executing and reading the index tests and the reference standard.	8–9
	11	Whether or not the readers of the index tests and reference standard were blind (masked) to the results of the other test and describe any other clinical information available to the readers.	N/A
Statistical methods	12	Methods for calculating or comparing measures of diagnostic accuracy, and the statistical methods used to quantify uncertainty (e.g., 95 % confidence intervals).	9, 10, Appendix 3
	13	Methods for calculating test reproducibility, if done.	N/A
Results			
Participants	14	Report when study was done, including beginning and ending dates of recruitment.	9, Additional file 1: Figure S1
	15	Report clinical and demographic characteristics of the study population (e.g., age, sex, spectrum of presenting symptoms, comorbidity, current treatments, recruitment centers).	10, Table 1
	16	Report the number of participants satisfying the criteria for inclusion that did or did not undergo the index tests and/or the reference standard; describe why participants failed to receive either test (a flow diagram is strongly recommended).	Additional file 1: Figure S1
Test results	17	Report time interval from the index tests to the reference standard, and any treatment administered between.	10
	18	Report distribution of severity of disease (define criteria) in those with the target condition; other diagnoses in participants without the target condition.	10, Table 1
	19	Report a cross tabulation of the results of the index tests (including indeterminate and missing results) by the results of the reference standard; for continuous results, the distribution of the test results by the results of the reference standard.	10–11, Table 2, Table 3
	20	Report any adverse events from performing the index tests or the reference standard.	N/A
Estimates	21	Report estimates of diagnostic accuracy and measures of statistical uncertainty (e.g., 95 % confidence intervals).	10–11, Table 2, Table 3, Additional file 1: Figure S2 and S3
	22	Report how indeterminate results, missing data, and outliers of the index tests were handled	N/A
	23	Report estimates of variability of diagnostic accuracy between subgroups of participants, readers or centers, if done	N/A
	24	Report estimates of test reproducibility, if done.	N/A
Discussion	25	Discuss the clinical applicability of the study findings	15

## Appendix 2

**Table 5:** Coding definitions for demographic, comorbid conditions, and AKI characteristics

Characteristic	Database	Codes
Age, sex, income, rural	RPDB	
Race, time on dialysis prior to transplant, date of kidney transplant, type of donor, cause of ESRD	CORR	
Diabetes mellitus	CIHI-DAD/OHIP	ICES derived cohort-ODD
Hypertension	CIHI-DAD/OHIP	ICES derived cohort-HYPERTENSION
Congestive heart failure	CIHI-DAD/OHIP	ICD9: 425, 5184, 514, 428
		ICD10: I500, I501, I509, I255, J81
		CCP: 4961, 4962, 4963, 4964
		CCI: 1HP53, 1HP55, 1HZ53GRFR, 1HZ53LAFR, 1HZ53SYFR
		OHIP fee codes: R701, R702, Z429
		OHIP diagnosis code: 428
Coronary artery disease	CIHI-DAD/OHIP	ICD9: 412, 410, 413, 414, 4292, 4295, 4296, 4297
		ICD10: I21, I22, Z955, Z958, Z959, R931, T822
		CCI: 1IJ26, 1IJ27, 1IJ50, 1IJ54, 1IJ57, 1IJ76
		CCP: 4801, 4802, 4803, 4804, 4805, 481, 482, 483
		OHIP: R741, R742, R743, G298, E646, E651, E652, E654, E655, G262, Z434, Z448
		OHIP diagnosis code: 410, 412, 413
Stroke/TIA	CIHI-DAD	ICD9: 430, 431, 4340, 4341, 4349, 436, 435, 3623
		ICD10: H341, I630, I631, I632, I633, I634, I635, I638, I639, G45, I629, I64, G45”, H340, I600, I601, I602, I603, I604, I605, I606, I607, I607, I609, I61
Chronic obstructive pulmonary disease	CIHI-DAD	ICD9: 491, 492, 496
		ICD10: J41, J43, J44
Chronic liver disease	CIHI-DAD/OHIP	ICD9: 4561, 4562, 070, 5722, 5723, 5724, 5728, 573, 7824, V026, 571, 2750, 2751, 7891, 7895
		ICD10: B16, B17, B18, B19, I85, R17, R18, R160, R162, B942, Z225, E831, E830, K70, K713, K714, K715, K717, K721, K729, K73, K74, K753, K754, K758, K759, K76, K77
		OHIP diagnosis code: 571, 573, 070
		OHIP fee code: Z551, Z554
Peripheral vascular disease	CIHI-DAD/OHIP	ICD9: 4402, 4408, 4409, 5571, 4439, 444
		ICD10: I700, I702, I708, I709, I731, I738, I739, K551
		CCP: 5125, 5129, 5014, 5016, 5018, 5028, 5038
		CCI: 1KA76, 1KA50, 1KE76, 1KG26, 1KG50, 1KG57, 1KG76MI, 1KG87
		OHIP feecode: R787, R780, R797, R804, R809, R875, R815, R936, R783, R784, R785, E626, R814, R786, R937, R860, R861, R855, R856, R933, R934, R791, E672, R794, E672, R813, R867, E649

*Abbreviations*: *RPDB* Registered Persons Database; *CIHI-DAD* Canadian Institute for Health Information Discharge Abstract Database; *OHIP* Ontario Health Insurance Plan; *CCP* Canadian Classification of Diagnostic, Therapeutic, and Surgical Procedures; *CCI* Canadian Classification of Interventions; *CORR* Canadian Organ Reporting Register; *TIA* transient ischemic attack; *AKI* acute kidney injury

## Appendix 3

**Table 6:** Sample 2 by 2 table for assessing diagnostic performance characteristics (sensitivity, specificity, positive predictive value, and negative predictive value) for *ICD-10* code N17x

	Reference standard: AKIN definition of AKI	
	≥2-fold increase in serum creatinine concentration from baseline	<2-fold increase in serum creatinine concentration from baseline
Code N17x positive	True positive (TP)	False positive (FP)
Code N17x negative	False negative (FN)	True negative (TN)

Sensitivity (Sn) = TP ÷ (TP + FN); the proportion of patients with ≥2-fold increase in serum creatinine concentration from baseline who are code N17x positive

Specificity (Sp) = TN ÷ (FP + TN); the proportion of patients with <2-fold increase in serum creatinine concentration from baseline who are code N17x negative

Positive predictive value (PPV) = TP ÷ (TP + FP); the proportion of patients who are code N17x positive with ≥2-fold increase in serum creatinine concentration from baseline

Negative predictive value (NPV) = TN ÷ (FN + TN); the proportion of patients who are code N17x negative with <2-fold increase in serum creatinine concentration from baseline

Positive likelihood ratio (LR+) = sensitivity/(1-specificity)

## Additional file

Additional file 1: Figure S1–S3. Patient Selection. Absolute changes in serum creatinine among patients who were code negative and code positive for AKI. Relative changes in serum creatinine among patients who were code negative and code positive for AKI. (DOCX 230 kb)

## Abbreviations

AKI: acute kidney injury
AKIN: acute kidney injury network
CIHI: Canadian Institute for Health Information
CKD: chronic kidney disease
ICD-10: International Classification of Diseases, Tenth Revision
ICES: Institute for Clinical Evaluative Sciences
PPV: positive predictive value
