# Supplementary material for: Validation of administrative database codes for acute kidney injury in kidney transplant recipients
Source: Can J Kidney Health Dis. 2016 Apr 7;3:18. doi: 10.1186/s40697-016-0108-7 (PMC4823855; doi:10.1186/s40697-016-0108-7)
Supplement: Additional file 1: Figure S1–S3. — Patient Selection. Absolute changes in serum creatinine among patients who were code negative and code positive for AKI. Relative changes in serum creatinine among patients who were code negative and code positive for AKI. (DOCX 230 kb) [file 40697_2016_108_MOESM1_ESM.docx]

**
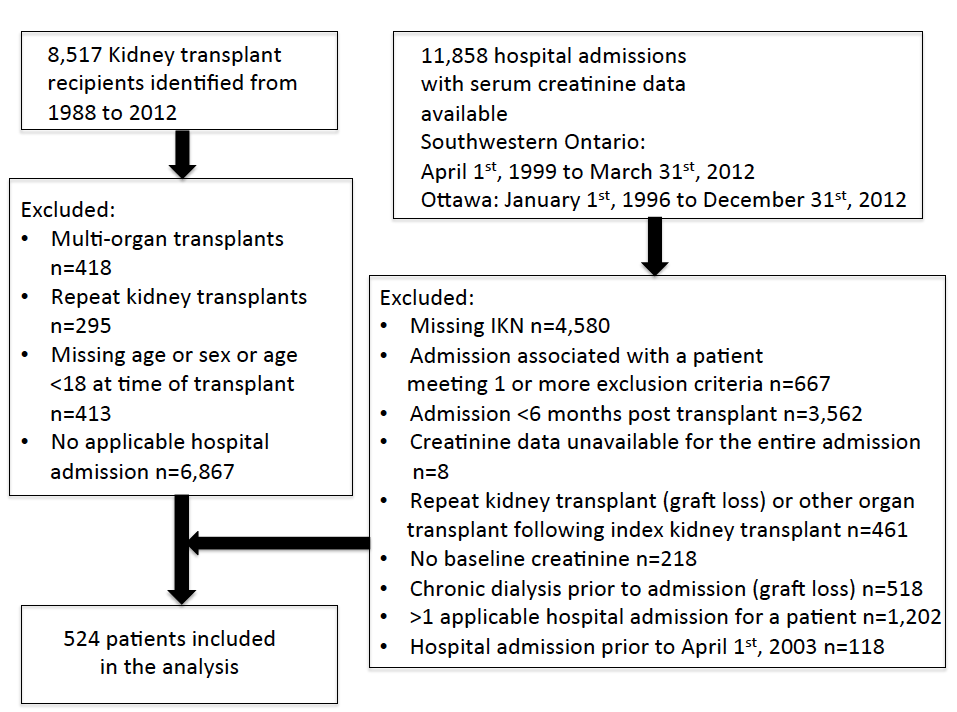
**

**Supplementary Figure 1: Patient Selection**


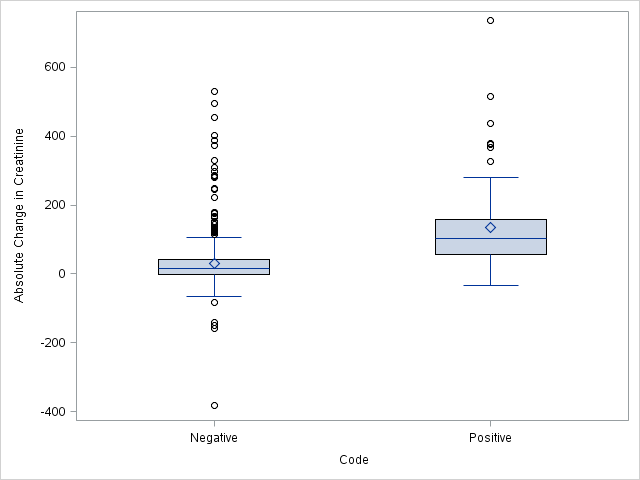


**Supplementary Figure 2: Absolute changes in serum creatinine among patients who were code negative and code positive for AKI.***

The boxes represent the interquartile range (50% of the values). The line across the box indicates the median. The diamond indicates the mean. The whiskers extend to the 95^th^ and 5^th^ percentiles.

*The *ICD-10* N17x code type considered is all diagnoses.


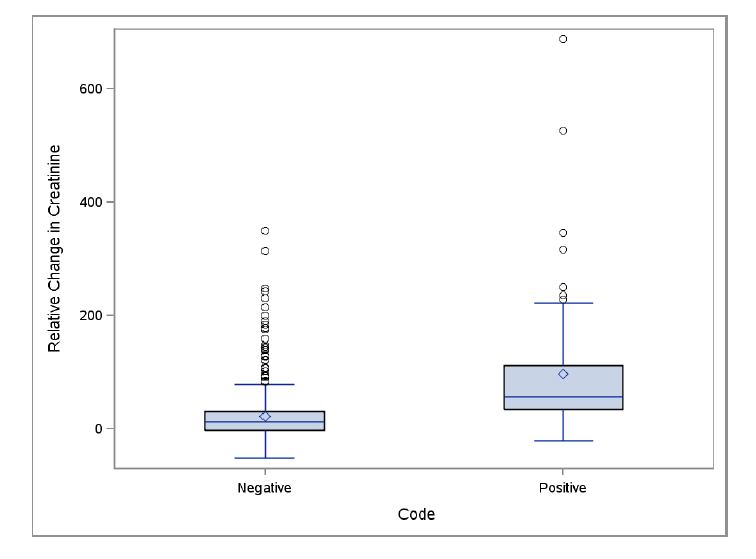


**Supplementary Figure 3: Relative changes in serum creatinine among patients who were code negative and code positive for AKI.***

The boxes represent the interquartile range (50% of the values). The line across the box indicates the median. The diamond indicates the mean. The whiskers extend to the 95^th^ and 5^th^ percentiles.

*The *ICD-10* N17x code type considered is all diagnoses.
